# Increased Inflammatory Response in Old Mice is Associated with More Severe Neuronal Injury at the Acute Stage of Ischemic Stroke

**DOI:** 10.14336/AD.2018.0205

**Published:** 2019-02-01

**Authors:** Fanxia Shen, Lidan Jiang, Frank Han, Vincent Degos, Shengdi Chen, Hua Su

**Affiliations:** ^1^Department of Neurology & Institute of Neurology, Rui Jin Hospital, Shanghai Jiao Tong University School of Medicine, Shanghai, China; ^2^Center for Cerebrovascular Research, Department of Anesthesia and Perioperative Care, University of California, San Francisco, CA 94143, USA.

**Keywords:** ischemic stroke, macrophage, blood-brain barrier, permeability, cytokine

## Abstract

Stroke occurs mostly in patients with advanced age. Elderly patients have a less favorable prognosis compared with young adult patients. To understand the underlying mechanisms, we tested our hypothesis that an increased inflammatory response to acute ischemic injury in old stroke mice leads to more severe brain damage and behavioral dysfunction. An ischemic stroke model was created in 2- and 12-month-old C57BL/6 mice through permanent occlusion of the left distal middle cerebral artery (dMCAO). Infarct/atrophy volumes were quantified by staining the brain sections with Cresyl Violet. Sensorimotor function was assessed using the corner test and adhesive removal test. Quantification of CD68^+^ cells in the peri-infarct region was performed at 1, 3 and 14 days after dMCAO. Interleukin-6 (IL-6), interleukin-1 β (IL-1β) and vascular endothelial growth factor (VEGF) levels in the ischemic brain tissue were measured using ELISA. Western blot was used to determine the expression levels of tight junction proteins, claudin-5 and zonula occludens (ZO)-1. Blood-brain barrier permeability was measured by Evans blue (EB) extravasation. Gelatinase B (MMP-9, type IV collagenase) was measured by gel zymography. Compared to 2-month-old mice, 12-month-old mice had more severe behavioral deficits at both the acute and chronic stages of stroke. Compared with the 2-month-old mice, 12-month-old mice had larger infarct/atrophy volumes at 1 and 14 days after dMCAO, higher levels of IL-6 and IL-1β, higher MMP9 activity, and lower levels of claudin-5 and ZO-1 at 1 and 3 days after dMCAO. 12-month-old mice also had more CD68^+^ cells in the peri-infarct region at 1, 3 and 14 days after dMCAO and more EB leakage at 3 days after dMCAO. A higher inflammatory response at the acute stage of ischemic stroke in old mice is associated with more severe neuronal injury and long-term behavioral dysfunction.

Age is a major risk factor for stroke. Not surprisingly, stroke has been reported to typically occur in the elderly [1]. In the United States, more than 66% of hospitalized stroke patients were over the age of 65 [2]. The elderly not only have a higher incidence of stroke but also less than optimal post-stroke recovery compared with their younger counterparts [3].

Aging is associated with a decline in cellular function and low-grade inflammation [4, 5]. Although the mechanisms are incompletely understood, it is well known that aging is associated with an increase of systemic inflammatory cytokines such as IL-1β and TNFα [6]. Interestingly, a number of pro-inflammatory cytokines such as IL-10 are implicated in neuroprotection [7, 8]. However, the role of inflammation in the ischemic brain remains unclear. Findings from experimental ischemic stroke models and clinical studies have demonstrated a significant contribution of inflammation to pathological features and symptoms of stroke [9, 10]. Inflammation appears to begin early after ischemic insult [11]. Yet, the age-dependent role of inflammation associated with increased ischemic brain injury has not been fully elucidated.

Most studies focusing on ischemic stroke have been using young healthy rodents as their models [12, 13]. However, the results obtained may not be translatable to elderly patients. Aging is associated with a series of processes, which involve systemic inflammation and metabolic dysfunction [14]. Normal aging is characterized by a chronic low-grade inflammatory state with a characteristic systemic increase of proinflammatory agents [15, 16]. To better understand the underlying mechanisms of how aging affects functional recovery of ischemic stroke victims, we examined the hypothesis that an increased inflammatory response to acute ischemic injury in old mice leads to more severe brain damage and a less favorable prognosis.

## MATERIALS AND METHODS

### Experimental groups

All experimental procedures involving animals were approved by the University of California, San Francisco Committee on Animal Research and conformed to the NIH Guidelines for the use of animals in research. C57BL/6 mice (Charles River, Burlington, MA) were used; 2-month-old mice were termed young while 12-month-old were known as old mice. The experimental groups are listed in Table 1.

### Animal stroke model

Animals were subjected to permanent occlusion of the left distal middle cerebral artery (dMCAO) [17]. Briefly, following anesthesia with 2% isoflurane inhalation, mice received a 1-cm incision between the left orbit and tragus. A piece of skull measuring 2 mm^2^ was removed and the left middle cerebral artery was permanently occluded by electrocoagulation (Fine Science Tools, Foster City, CA, USA). Body temperature was maintained at 37 ± 0.5 °C using a thermal blanket throughout the surgical procedure. Surface cerebral blood flow (CBF) was monitored by a laser Doppler flow meter (Vasamedics Inc, Little Canada, MN, USA) during stroke induction. Mice with CBF that was more than 15% of the baseline in the ischemic core after dMCAO were excluded from the experiment. Animals were sacrificed after 1, 3 and 14 days where their brain samples were collected for further analyses.

**Table 1:** Experimental groups used in this study.

Days post-dMCAO	Age	
**D1**	Young (18 mice)	Old (18 mice)
**D3**	Young (18 mice)	Old (18 mice)
**D14**	Young (12 mice)	Old (12 mice)

### Assessment of behavioral deficits

Neurobehavioral outcomes were examined through the adhesive removal test and corner test [18]. All animals underwent behavioral tests at 3 and 14 days after dMCAO. Mice were trained for 3 days before dMCAO with 3 consecutive trials to generate stable baseline values.

1. The adhesive removal test was used to assess somatosensory deficits as previously described. Mice were placed in the testing box. After 60 seconds of habituation period, adhesive tapes (0.3 × 0.3 cm) were applied to both forepaws with equal pressure. The time to remove the tape from each paw was assessed with a maximum testing time of 120 seconds.
2. The corner test was used to detect sensorimotor and postural asymmetries. As previously described, two identical boards with dimensions 30 × 20 cm^2^ were put together to form a 30° angle. Mice were placed between the boards. When entering deep into the corner, both sides of their vibrissae were stimulated. The mouse rears forward and upward, and then turns back to face the open end. Normal mice would turn either to the left or right side with equal frequency while mice subjected to ischemic stroke would turn more often to the lesion side (in this study, this will be the left side). The turns were recorded from 10 trials for each test. Turns that were not part of a rearing movement were not scored. Data are presented as normalized turn ratio out of 10 trials.

### Tissue Processing

Mice were euthanized at 1, 3 and 14 days post dMCAO. Brain tissues were collected. For zymography, ELISA and Weston blot analyses, the protein was extracted from the peri-ischemic area by homogenizing brain tissue in phosphate-buffered saline containing protease inhibitor (Pierce Biotechnology Inc, Rockford, IL, USA) and quantified using the BCA protein assay kit (Pierce Biotechnology Inc, Rockford, IL, USA).

### Evaluation of infarct and atrophy volume

Infarct volume was evaluated using a protocol adapted from previously published methods [19]. Briefly, serial coronal sections, 20 μm in thickness and 200 μm in interval from the frontal pole were prepared using a cryostat. Cresyl violet staining was used to identify the infarct area one day post dMCAO and atrophy area 14 days post dMCAO. The area of infarct/atrophy and non-infarct tissues were outlined using NIH’s Image J analysis system.

The infarct volumes were reconstructed by taking the sum of infarct areas from all sections and multiplying by 200 µm. To avoid the influence of edema at 1 day after stroke, the ratio of infarct volume versus ipsilateral hemisphere volume was calculated as described in our previously published paper [18].

The atrophic area was calculated as the area of the normal area of the ischemic hemisphere subtracted from the non-ischemic hemisphere. The ratio of atrophic volume versus ipsilateral hemisphere volume was then calculated.

### Enzyme-linked immunosorbent assay (ELISA)

At either 1 or 3 days after dMCAO, IL-6, IL-1β and VEGF levels in the ischemic brain tissue were measured using ELISA kits (R&D Systems, Minneapolis, MN, USA) following the manufacturer’s instructions. The plates were read using a micro-titer plate reader spectrophotometer (GENESYS 10S UV-Vis Spectrophotometer; Thermo Electron Corp, Madison, WI, USA).

### Analysis of brain-blood barrier (BBB) permeability

BBB permeability was evaluated by Evans Blue (EB) dye exudation. EB extravasations were performed using the method described previously [19, 20]. With animals under anesthesia, 2% Evans blue dying (30 mg/kg) was infused through the jugular vein and allowed to circulate for 60 min. The brains were then removed and divided into right hemisphere and left hemisphere. Brain samples were homogenized in formamide. After incubating the homogenized brain samples overnight at 55 °C, the samples were centrifuged at 12,000 RPM for 45 min. The supernatant was used to measure the absorbance of Evans blue at 620 nm using a spectrophotometer (GENESYS 10S UV-Vis Spectrophotometer; Thermo Electron Corporation, Madison, WI). Evans blue content was expressed as micrograms per hemisphere, which was calculated against a standard. To control the variations of perfusion efficiency, the absorbance of the contralateral hemisphere was subtracted from that of the hemisphere ipsilateral to the dMCAO.

### Gelatin zymography

Zymography was performed as previously described [21]. Briefly, 30 μg protein from each sample was separated under nonreducing conditions in a 10% zymogram gel (Invitrogen, Carlsbad, CA, USA) as indicated by the manufacturer’s instructions. After electrophoresis, the gel was washed and incubated in the development solution overnight at 37 °C and then stained with 0.5% Coomassie blue. MMP-9 activity can be detected as white bands of lysis against the Coomassie blue stained gel. Protein bands in zymography were quantified by scanning densitometry using Kodak image analysis software (Eastman Kodak Company, Rochester, NY, USA).

### Immunohistochemical analysis

Immunohistochemical staining was performed as described [19]. Briefly, sections were incubated with primary antibodies at the following concentrations: mouse anti-CD68 (1:100, AbD Serotec, Raleigh, NC, USA) at 4 °C overnight and then incubated with Alexa Fluor 488-conjugated IgG (Molecular Probes, Carlsbad, CA, USA) at a dilution of 1:300.

### Cell counting

CD68^+^ cells in the ischemic region were quantified for brain sections collected 1, 3 and 14 days after dMCAO as described previously [17]. Cell counting was performed separately by two blinded investigators using Image J.

### Western blotting

Western blot was performed as previously reported [19]. An equal amount of protein isolated from brain samples were loaded on a 4-12% Bis-Tris gel. Following electrophoresis, the proteins were electroblotted onto PVDF membranes. The membranes were then probed with antibodies specific to ZO-1 and claudin-5 (1:200, Invitrogen, Grand Island, NY, USA) followed by appropriate species-specific IRDye® secondary antibodies (1:10000, Li-Cor Biotechnology, Lincoln, NE, USA). GAPDH (1:10000; Sigma-Aldrich, St. Louis, MO, USA) was used as a loading control. PVDF membrane was scanned at both 700 and 800 nm channels using an Odyssey CLx System (Lincoln, Nebraska, USA) [22, 23]. Results are expressed as a relative density to GAPDH.

### Statistical analysis

Data are presented as mean ± standard deviation. Parametric data obtained from the older and younger mice were compared using one-way analysis of variance (ANOVA), followed by Fisher’s protected least significant difference (PLSD) test. A probability value <0.05 was considered statistically significant. Sample sizes for each analysis are indicated in figure legends.

**Figure 1.**
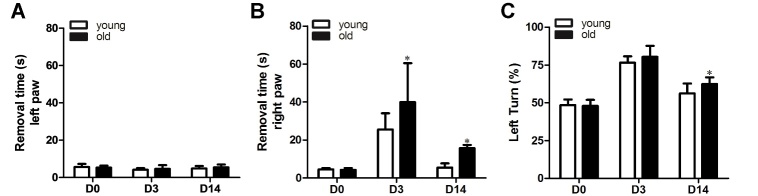
**Old mice had more severe functional deficits at both the acute and chronic stages of dMCAO**. A) Quantification of the time mice used to remove tapes from their left paws. B) Quantification of the time mice used to remove tapes from their right paws. Old mice tended to spend more time to remove the tape on their right paw at 3 and 14 days after dMCAO (*p<0.05, old vs. young). C) Quantification of left turn in corner test. No significant difference was observed between old and young mice at 3 days after dMCAO. Old mice, however, showed more frequent turning to the lesion side at 14 days after dMCAO (*p=0.02, old vs. young). Data represented as mean ± SD, n=10.

## RESULTS

### Old mice had more severe functional deficits than young mice after ischemic stroke

First, we investigated functional recovery after focal cerebral ischemia using the adhesive removal test and corner test. The adhesive removal test showed that both the 2 and 12-month-old mice took a longer time to remove the tape from their right paw (affected paw) 3 days after dMCAO (old vs. baseline: 39.5±19.5 seconds [s] vs. 4.3±1.0 s, p<0.001, young vs. baseline: 27.7±8.1 s vs. 4.6±0.6 s, p<0.001) while this time was reduced at 14 days after dMCAO (old vs. baseline: 15.3±1.6 s, p=0.008, and young vs. baseline: 5.5±1.5 s, p=0.97, Fig. 1). Interestingly, age alone did not influence baseline performance (old vs. young: 4.3±1.0 s vs. 4.6±0.6 s, p>0.9). However, the young mice used a significantly shorter time to remove the tape on their right paw than the older mice at both 3 (p=0.012) and 14 (p=0.04) days post dMCAO. Adhesive removal from the left paw was not affected (Figs. 1A and B).

For the corner test, both young and old mice turned more frequently to the lesion side 3 days (old vs. baseline: 80.4±7% vs. 47.9±4%, p<0.001; young vs. baseline: 76.6±4% vs. 48.5±4%, p<0.01) and 14 days (old vs. baseline: 62.4±5%, p<0.001, young vs. baseline: 56.6±7%, p<0.001) after dMCAO. No difference was detected between young and old mice at 3 days after dMCAO (p=0.3). At 14 days after dMCAO, the old mice made more left turns than the young mice (p=0.03, Fig. 1C). These data suggest that functional recovery of the old mice was slower compared with the young mice.

### Old mice had larger infarct/atrophy volumes

**Figure 2.**
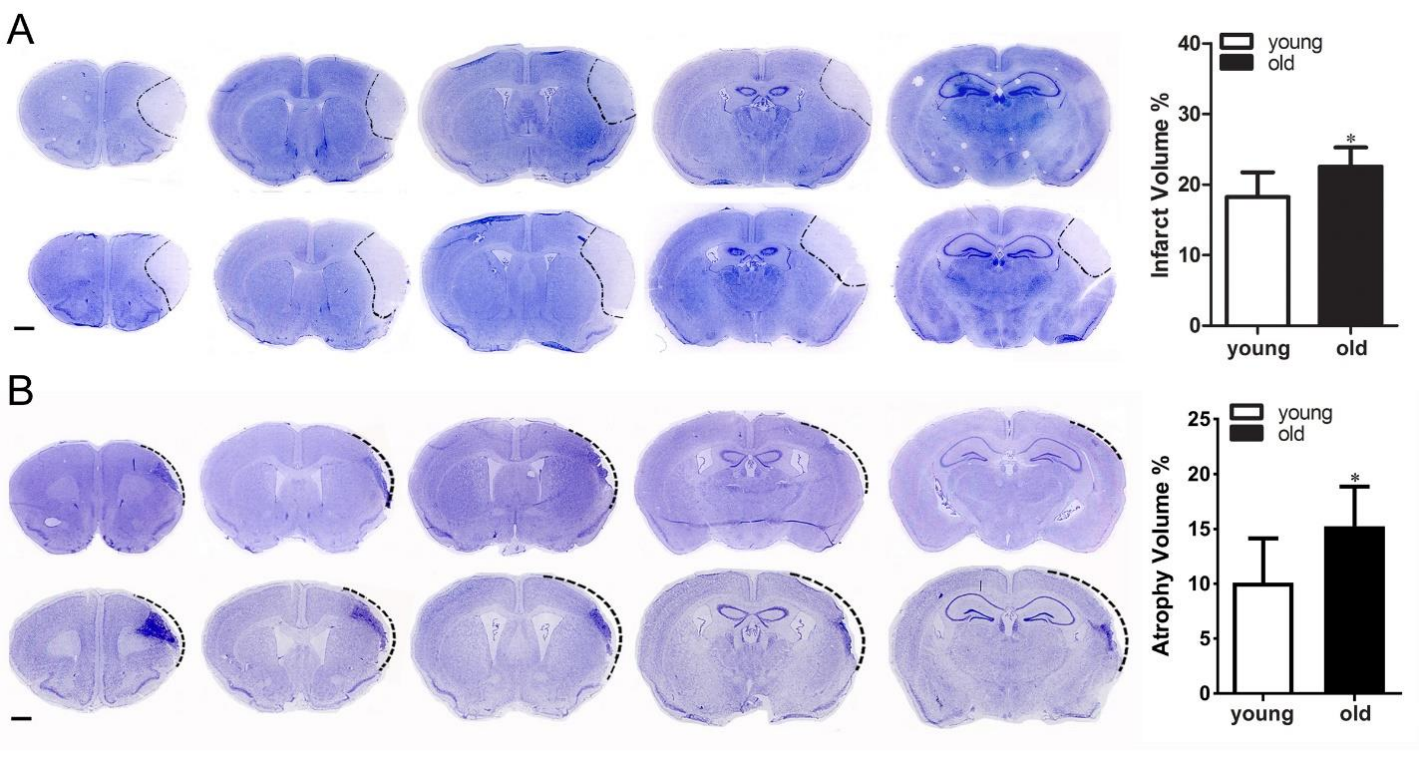
**Old mice had larger infarct/atrophy volumes**. A) Representative Nissl-stained brain coronal sections (left) and bar graph (right) show infarct volume was larger in old mice than in young mice (*p= 0.04) at 1-day post dMCAO. B) Nissl-stained sections (left) and bar graph (right) show that old mice have significantly larger atrophic volume than young mice 14 days after dMCAO (*p<0.05). Data represented as mean ± SD, n=8.

Through analyzing cresyl violet stained sections, we found that compared with young mice (17.63±3.89% of total volume), the old mice (22.29±3.04%) had larger infarct volumes at day 1 after dMCAO (p=0.02, Fig. 2A). The old mice (15±4 % of total volume) also had lager atrophy volumes than the young mice (10±4%) at day 14 after dMCAO (p=0.02, Fig. 2B).

### Old mice had more CD68 positive cells around the peri-infarct area

**Figure 3.**
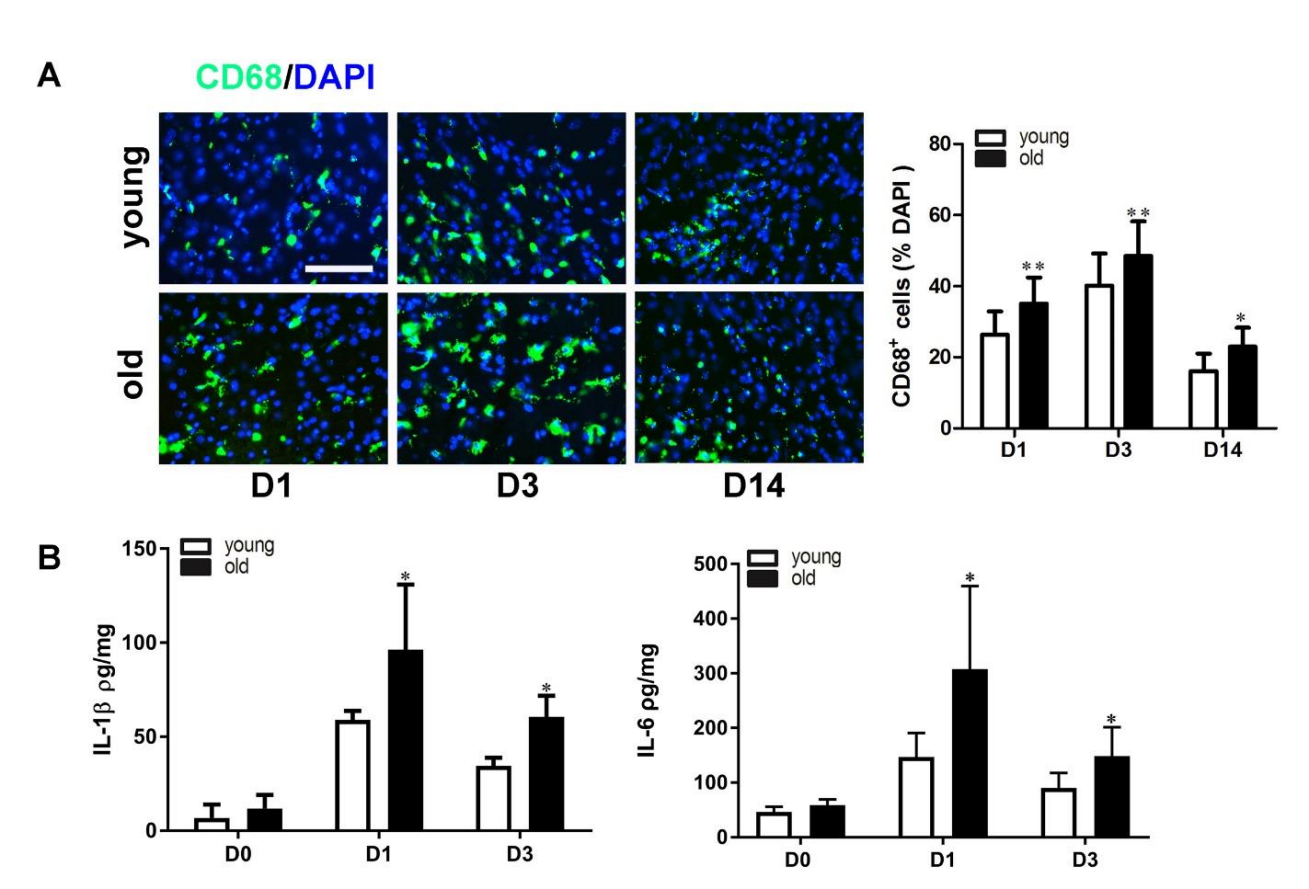
**Old mice had increased numbers of inflammatory cells and pro-inflammatory cytokines in the infarct area**. A) Representative images of immunofluorescent staining of CD68 (green) demonstrate that old mice had more CD68^+^ cells in the peri-infarct area at 1, 3 and 14 days after dMCAO. The bar graph shows quantification of CD68 positive cells. **p<0.01, *p<0.05 vs. young mice at the corresponding time points. n=6, bar=100 µm. B) Bar graphs show quantification of IL-1β (left) and IL-6 (right) protein levels in the ischemic brain tissue 1 and 3 days after dMCAO. Old mice had higher IL-1β and IL-6 levels at 1 day and up to 3 days after dMCAO. *p<0.05, n=6. Data represented as mean ± SD.

Macrophage/microglia activation is an indicator of neuroinflammation. CD68 is a marker for macrophages and activated microglia. Therefore, we quantified the number of CD68^+^ cells in the peri-infarct region (Fig. 3A).

CD68^+^ cells were detected at 1 day after ischemic stroke in the brains of both old and young mice, which peaked at 3 days, and were still present at 14 days after dMCAO. Compared with the young mice, the old mice had more CD68^+^ cells in the peri-infarct region at 1 day (old vs. young: 35.1±7.3% of total DAPI positive cells vs. 26.4±6%, p=0.0006), 3 days (old vs. young: 47.3±8.4% vs. 40.1±8.9%, p=0.006) and 14 days (old vs. young: 19.4±5.0% vs. 13.4±3.5%, p=0.032) after ischemic stroke (Fig. 3A). These data suggest that stroke-induced inflammation increases with aging.

### Old mice had high levels of pro-inflammatory cytokine expression in the ischemic brain

We next evaluated the IL-1β and IL-6 protein levels in the ischemic brain tissue and found that the levels of IL-1β (old vs. young 11.8±7 vs. 6.8±7 pg/mg, p=0.24) and IL-6 (old vs. young: 50±2 vs. 66±5 pg/mg, p=0.06,) were similar in normal old and young mice.

IL-1β and IL-6 increased at 1 day and 3 days after ischemic stroke in both groups, with a peak at 1 day after ischemic stroke (Fig. 3B). Compared with the young mice, the old mice had higher IL-1β and IL-6 protein levels at both 1 day (old vs. young IL-6: 308±152 pg/mg vs. 147±44, p=0.03; IL-1β: 96±35 vs. 55±8 pg/mg, p=0.0008) and 3 days (IL-6: 149±53 vs. 90±27, p=0.04; IL-1β: 61±17 vs. 54±8, p=0.02; old vs. young) after ischemic stroke (Fig. 3B).

### Old mice showed more severe blood-brain barrier leakage

**Figure 4.**
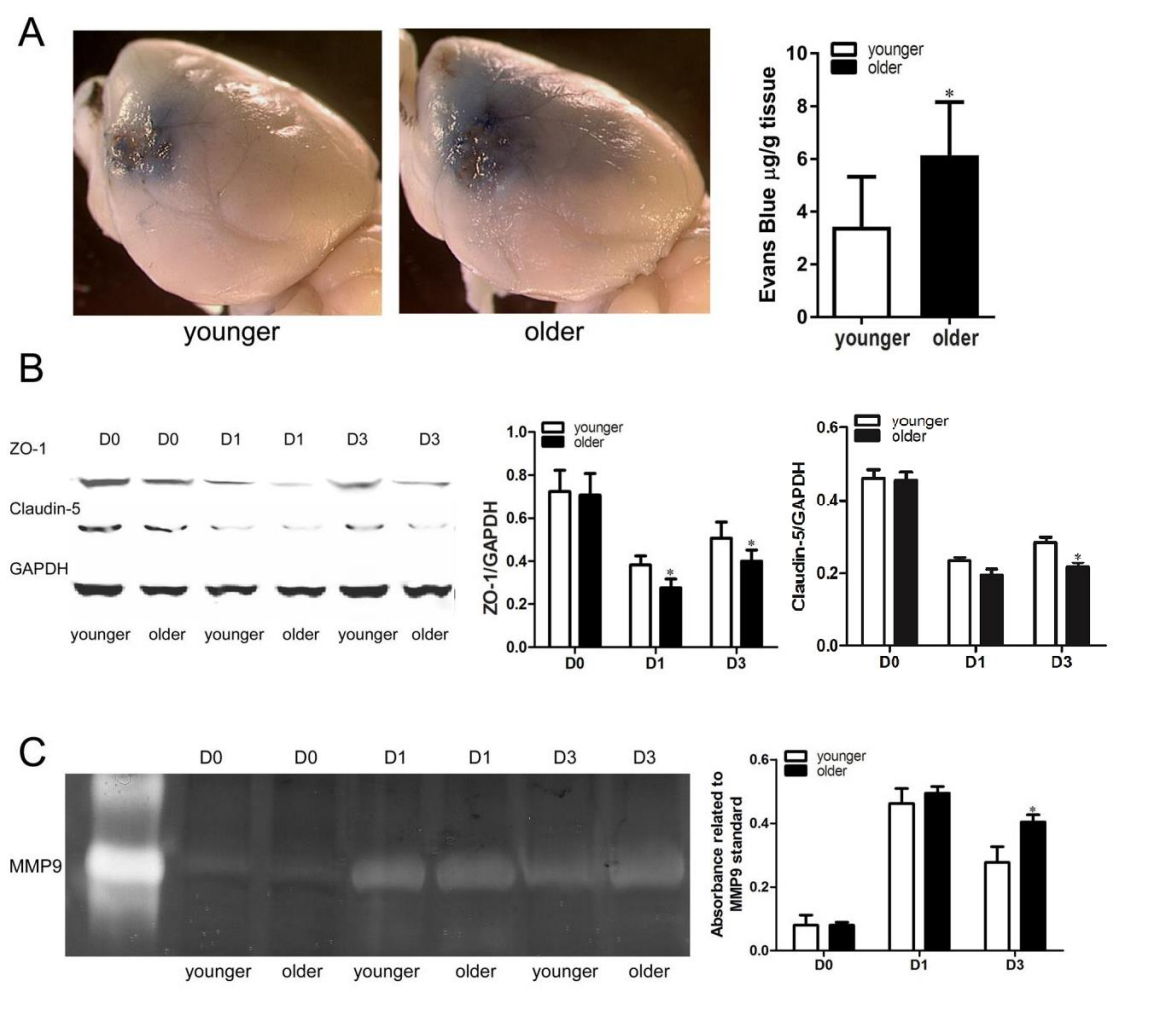
**Old mice showed more severe blood-brain barrier leakage**. A) Representative images of mouse brains perfused with Evans Blue dye. Old mice had more Evans blue dye on the brain tissue around the infarct site than young mice 3 days after dMCAO. The bar graph shows quantification of Evans blue presence (*p=0.04, vs. young), n=6. B) Representative images of western blot and bar graphs show quantification of ZO-1 (middle) and Claudin-5 (right) protein levels. Old mice express a lower level of ZO-1 than young mice at 1 and 3 days after dMCAO (*p<0.05, vs. young). Old mice also express a lower level of Claudin-5 than young mice at 3 days after dMCAO (*p<0.001, vs. young), n=6. C. Representative image of zymogram gel. Bar graph show elevation of MMP-9 at 3 days after dMCAO. (*p=0.001, vs. young), n=6. Data represented as mean ± SD.

Evans blue (EB) dye extravasation was used to assess BBB permeability. More EB dye was visualized on the brain surface of the infarct area than non-infarct brain area (Fig. 4A). More EB dye extravasated from the ipsilateral hemisphere of old mice (6.0±2.6/mg/g brain tissue) when compared with the young mice (2.9±1.8 µg/g, p=0.04, Fig. 4A).

Tight junction proteins including claudins-5 and ZO-1 in ischemic brain tissue were determined using western blot analysis (Fig. 4B). Levels of ZO-1 and claudins-5 were reduced at 1 and 3 days post dMCAO in both groups (p<0.0001, Fig. 4B). The old mice expressed lower levels of ZO-1 than the young mice at 1 day (old vs. young: 0.27±0.04 vs. 0.38±0.04, p=0.048) and 3 days (old vs. young: 0.39±0.05 vs. 0.51±0.08, p=0.039) post dMCAO. Although both groups had similar levels of claudin-5 at 1-day post dMCAO (old vs. young: 0.19±0.03 vs. 0.23±0.02, p=0.32), at 3 days post-ischemia, the old group had a lower level of claudin-5 than the young group (old vs. young: 0.22±0.03 vs. 0.3±0.05, p=0.0096, Fig. 4B).

The baseline expression level of claudins-5 and ZO-1 were similar between the two groups (old vs. young: 0.71±0.1 vs. 0.72±0.09, p=0.97).

MMP9 is a key component contributing to BBB damage [24]. Thus, we tested if aging affects MMP activity in ischemic brains using gel zymography. MMP-9 activity peaked at 1 day after dMCAO and decreased at 3 days in the ischemic brain (Fig. 4C). Both groups had similar levels of MMP9 at 1 day after dMCAO (old vs. young: 50±7 vs. 45±5% of MMP9 standard, p=0.41). However, at 3 days after dMCAO, old mice had a higher level of MMP9 (40±7) than the young mice (28±5%, p=0.001). Our finding indicates that the aging process increases MMP activity in response to ischemic injury. At baseline, both groups had similar MMP9 activity (old vs. young: 8±3 vs. 8±2%, p=0.96).

### Old mice expressed lower level of VEGF after ischemic stroke

**Figure 5.**
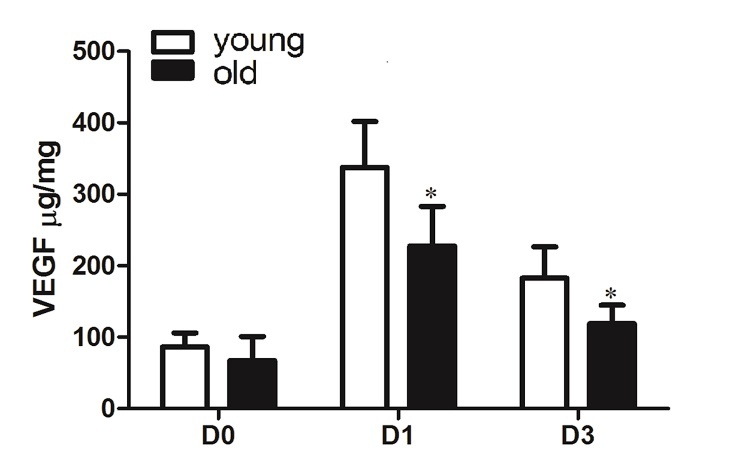
**Old mice had lower VEGF level in the ischemic brain tissue**. Bar graphs show ELISA assay of VEGF levels in ischemic brain tissues at 1 and 3 days after dMCAO. Compared to the young mice, old mice had lower average VEGF protein levels at 1 and 3 days after dMCAO (*p<0.05, vs. young), n=6. Data represented as mean ± SD.

The old mice had lower VEGF protein levels compared with their younger counterparts at 1 day (old vs. young: 228±56 vs. 338±64 µg/mg, p<0.001) and 3 days (old vs. young: 119±26 vs. 183±44µg/mg, p=0.049) after dMCAO (Fig. 5). VEGF protein levels in the normal brain were similar for both groups (old vs. young: 67± 34 vs. 87±19, p>0.9).

## DISCUSSION

In this study, we demonstrated that neuronal damage is more severe in 12-month-old mice after ischemic stroke than in 2-month-old mice, which could be attributed to the increased number of inflammatory cells and levels of pro-inflammatory cytokines. We also showed that 12-month-old mice had worse functional recovery than that of 2-month-old mice.

Much of the published studies suggest that inflammation increases stroke risk and contributes to the progression of the ischemic lesion. Microglia, which plays a critical role in regulating brain immunity, is known to be activated after brain ischemia [25].

CD68, a lysosomal protein is considered an indicator of phagocytic activity [26, 27]. Macrophages/microglia do not express CD68 in the healthy brain but become CD68-positive after acute ischemic stroke and/or intracerebral hemorrhage [28-30].

Consistent with a previously published study [31], we found that the number of CD68^+^ cells in the border zone of the ischemic area was greatly increased. We also demonstrated that the old mice had more CD68^+^ cells than the young mice after ischemic stroke.

Considering that the CD68 glycoprotein modulates the phagocytic activity of macrophages, more CD68^+^ macrophages present in the ischemic area of old mice brains could be related to more brain tissue damage.

CD68^+^ macrophages could be derived from peripheral blood. The infiltration of peripheral blood-derived CD68^+^ macrophages facilitated by the disruption of BBB integrity following brain ischemic injury could contribute to the neuroinflammation process [32]. The numbers of CD68^+^ cells were significantly increased in the peri-infarct area in old mice 2 weeks after ischemic stroke, indicating that aging might impair the resolution of inflammation. Activated macrophages could also produce pro-inflammatory cytokines, IL-1β and IL-6, thus exacerbating neuronal injury [33].

Aging is associated with alterations in the neuroinflammatory environment. Thus, age might be an important intrinsic factor determining the level of microglia activation as well as the production of inflammatory cytokines such as IL-1β and IL-6 after stroke. We show that compared with 2-month-old mice, the 12-month-old mice had reduced functional recovery after ischemic stroke, which was associated with the increase in the number of CD68^+^ macrophage/microglia, and higher inflammatory cytokines levels. Our study indicates that age increases proinflammatory responses in the ischemic brain.

IL-1β is one of the most prominent pro-inflammatory cytokines [34]. It has been shown that the expression of TNFα, IL-1β, and IL-6 increased in the rat brain’s cortex and striatum during aging, and the expression of these cytokines was mostly attributed to astrocytes, but not to microglia or neurons [35]. Circulating IL-6 level increases with age and increased IL-6 in circulation is a risk factor for various diseases and mortality [36]. Pro-inflammatory cytokines including IL-1β and IL-6 are elevated in the ischemic stroke brain, which may cause morphological and functional changes in the constituent cell types of the brain [37]. IL-1β is thought to have a negative effect on stroke recovery. Microglia are the major source of IL-1β after dMCAO [38, 39]. Our data indicate that the levels of inflammatory cytokines, IL-6 and IL-1β in the ischemic tissue are negatively correlated with neurological outcome. Old mice had higher levels of IL-6 and IL-1β in the ischemic tissue and thus, a worse outcome than the young mice. Furthermore, the levels of the inflammatory and anti-inflammatory cytokines such as IL-1β, IL-6, and IL-10, at the early stage of stroke might serve as important biomarkers to predict the prognosis for ischemic stroke patients.

IL-1β and IL-6 can modulate BBB properties. BBB disruption could result in more inflammatory cells infiltrating into the ischemic area. Still, relatively little is known about the changes of inflammation and BBB leakage during aging in response to ischemic brain injury. We proceeded to use three different methods to qualitatively evaluate BBB permeability after ischemic stroke, including Evans blue extravasation, which has been used for BBB integrity assessment as early as the 1960s [40]. Our results indicate that old mice had more dye present in the ischemic area compared with the young mice.

MMP-9, a member belonging to the family of matrix metalloproteinases (MMPs), has been suggested to be involved in the BBB breakdown in neurological disorders, including stroke [41, 42]. The high levels of MMPs can damage neurovascular matrix and cause BBB breakdown and brain edema. Increased activity of MMPs is thought to be a contributor to cell death and BBB disruption in the early stage of stroke [43]. Activated MMPs can degrade endothelial junctional proteins and the ECM [44]. MMP-9-independent BBB leakage shortly after acute stroke was thought to be a link between subtle early hyperpermeability and subsequent full-blown BBB degradation, parenchymal destruction and long-term neurological dysfunction [45]. It was evident that two peaks of BBB permeability appeared at 3 hours and 72 hours of reperfusion after 2 hours focal ischemia [46]. Also, MMP-9 activity during the delayed neuroinflammatory response may contribute to remodeling and stroke recovery, but MMP-9 activity in the acute stroke stage can exacerbate BBB leakage as well as brain damage [47]. In our study, MMP-9 activation and BBB leakage appear to coincide with increased inflammatory cytokine expression and brain damage severity.

To further confirm the impairment of BBB after ischemic stroke, we evaluated the levels of tight junction protein complexes namely, claudin-5 and ZO-1. Tight junction proteins are important structural components of the BBB [48]. Reduced expression of tight junction proteins has been associated with BBB dysfunction in a number of disorders including ischemic stroke [49, 50]. In this study, we demonstrated that the reduction of claudin-5 and ZO-1 were correlated with the degree of BBB leakage, ischemic brain injury, and neurological dysfunction. The claudin-5 level was similar to the normal brains of 2- and 12-month-old mice, which suggests that age could have caused changes that are more prominent under disease conditions.

VEGF is an angiogenic growth factor that has a neuroprotective effect [51]. We have previously demonstrated that VEGF receptor 2 expression was reduced in aged mice [18]. Here, we showed that VEGF level was reduced in the ischemic brain tissue of old mice, which indicates that impaired angiogenesis might be correlated with worse outcome in older mice after ischemic stroke.

In summary, we showed that a higher inflammatory response in the acute stage of ischemic stroke in old mice was associated with more severe neuronal injury (larger infarct volume) and long-term functional deficits. The mechanisms responsible for the enhanced inflammatory response will require additional studies. However, modulating inflammatory response at the acute stage of stroke might be a strategy to reduce neuronal injury and improve functional recovery in elderly stroke patients.
